# Polymorphism rs4919510:C>G in Mature Sequence of Human MicroRNA-608 Contributes to the Risk of HER2-Positive Breast Cancer but Not Other Subtypes

**DOI:** 10.1371/journal.pone.0035252

**Published:** 2012-05-07

**Authors:** A-Ji Huang, Ke-Da Yu, Jing Li, Lei Fan, Zhi-Ming Shao

**Affiliations:** Department of Breast Surgery, Cancer Center and Cancer Institute, Shanghai Medical College, Fudan University, Shanghai, People’s Republic of China; IFOM, Fondazione Istituto FIRC di Oncologia Molecolare, Italy

## Abstract

**Background:**

A few polymorphisms are located in the mature microRNA sequences. Such polymorphisms could directly affect the binding of microRNA to hundreds of target mRNAs. It remains unknown whether rs4919510:C>G located in the mature miR-608 alters breast cancer susceptibility.

**Methods:**

The association of rs4919510:C>G with risk and pathologic features of breast cancer were investigated in two independent case-control studies, the first set including 1,138 sporadic breast cancer patients (including 927 invasive ductal carcinoma patients, 777 of them with known subtypes: 496 luminal-like, 133 HER2-positive, and 148 triple-negative) and 1,434 community-based controls, and the second set including 294 familial/early-onset breast cancer patients and 500 hospital-based cancer-free controls. Odds ratios (ORs) were estimated by logistic regression. Predicted targets of miR-608 and complementary sequences containing rs4919510:C>G were surveyed to reveal potential pathological mechanism.

**Results:**

In the first set, although rs4919510:C>G was unrelated to breast cancer in general patients, variant genotypes (CG/GG) were specifically associated with increased risk of HER2-positive subtype (Adjusted OR = 1.97, 95% CI, 1.34−2.90 in the recessive model). Variant G-allele was the risk allele with OR of 1.62 (95% CI, 1.23−2.15). Patients carrying GG-genotype also had larger HER2-positive tumors (P for Kruskal-Wallis test = 0.006). The relationship between rs4919510:C>G and risk of HER2-positive subgroup was validated in the second set (Bonferroni corrected P = 0.06). The adjusted combined OR (total 164 HER2-positive cases) in the recessive model was 1.97 (95% CI, 1.43−2.72) for GG genotype (corrected P = 1.1×10^−4^). Bioinformatic analysis indicated that, HSF1, which is required for HER2-induced tumorigenesis, might be a target of miR-608. The minimum free-energy of ancestral-miR-608 (C-allele) binding to HSF1 is −35.9 kcal/mol, while that of variant-form (G-allele) is −31.5 kcal/mol, indicating a lower affinity of variant-miR-608 to HSF1 mRNA.

**Conclusion:**

rs4919510:C>G in mature miR-608 may influence HER2-positive breast cancer risk and tumor proliferation.

## Introduction

MicroRNAs (miRNAs) are an abundant class of small non-protein-coding RNAs that act as negative gene-regulators. miRNAs represent ideal candidates for cancer predisposition loci because small variation in quantity has an effect on hundreds even thousands of target mRNAs and might result in diverse functional consequences [1], [2]. A strong link between altered miRNAs, either in structure or in quantity of mature product, and various cancer risks has been established. Genetic variants such as single-nucleotide polymorphisms (SNPs) and mutations may change the property of miRNAs through altering miRNA expression and/or maturation.

The role of genetic variants in miRNAs or in miRNA-targeting sites in breast cancer susceptibility has attracted much attention. Several SNPs in the sequences of pre-miRNAs such as miR-196a2 (rs11614913:T>C), miR–499 (rs3746444:A>G), and miR-125a (rs12975333:G>T) were associated with significantly increased risks of breast cancer in some but not all studies [2], [3], [4].

Because SNPs located in the mature miRNA region could directly affect the binding to target mRNAs, we focused on this kind of SNPs for molecular epidemiological study. There are only a few such SNPs according to bioinformatics survey (i.e., rs12975333 in miR-125a, rs4822739 in miR-548j, and rs4919510 in miR-608). rs4919510:C>G in mature miR-608 is of particular interests because it is predicted that its variant form can bind with a different energy to its targets. For example, the ancestral form of miR-608 binds its target within the insulin receptor (INSR) mRNA with a △G of −24.04 kJ/mol, whereas its variant form binds with a free energy of -19.17 kJ/mol [5]. Moreover, the predicted targets of miR-608 include interleukin-1 alpha (IL1A), growth hormone receptor (GHR), and TP53 [5]. Of note, INSR [6], IL1A [7], GHR [8], and TP53 [9] were reported to be associated with breast cancer. Therefore, we hypothesized that rs4919510:C>G in mature miR-608 might relate to breast cancer. To test hypothesis, we genotyped this SNP and evaluated its association with breast cancer risk as well as clinical features in two independent case-control sets of Chinese women, totally comprising 1,432 breast cancer cases and 1,934 cancer-free controls.

## Materials and Methods

### Patients

In the first set, all the participants were genetically unrelated Han Chinese women living in Shanghai City and its surrounding areas [10]. The 1,138 patients had pathologically-confirmed primary breast cancer and were consecutively recruited from the Department of Breast Surgery at Fudan University Shanghai Cancer Center (FUSCC) between January 2006 and December 2008. Participants with a previous history of cancer (except breast cancer) and metastatic breast cancer were excluded. The 1,434 controls were from a community-based breast cancer screening program as previously described [11]. All the controls were determined as cancer-free after comprehensive examinations. After finishing a written informed consent document, each participant was carefully interviewed to obtain epidemiological information and donated approximately 3−5 ml of peripheral venous blood. **Table 1** presents the characteristics of study subjects. Cases and controls were comparable in age (both median age was 49 years, P = 0.761) and menopausal status (42% postmenopausal in both groups, P = 0.108). Compared with the controls, more women in patient group had a family history of first-degree relatives with breast cancer (2.7% versus 7.5%, P = 4.6×10^−8^). Among the 1,138 cases, 927 (81.5%) were invasive ductal carcinoma (IDC), 14.4% were ductal carcinoma *in situ* (DCIS), and 4.1% were other special histological types. IDC was classified as three subtypes according to the immunohistochemistry (IHC) status of estrogen receptor (ER), progesterone receptor (PR), and human epidermal growth factor receptor-2 (HER2). HER2 positivity was determined by IHC 3+ (HerceptTest, DAKO, Denmark) or fluorescence *in situ* hybridization (FISH) positive status (PathVysion HER2 DNA probe kit). Most of, but not all, patients with equal HER2 protein expression (IHC 2+) were also selected to have a FISH test for HER2 gene amplification. We defined subtypes as following: luminal-like (ER+ and/or PR+ and HER2-), triple-negative (ER-, PR-, and HER2-), and HER2+ (HER2+, regardless of ER/PR) [12].

**Table 1:** Summary characteristics of the participants in the first set.

				Cases (n = 1,138)	Controls (n = 1,434)	P
Age (median)				49 years	49 years	0.761
Age at menarche (median)				15 years	16 years	0.020
BMI (mean)				23.6	23.2	0.073.
Menopause (%)	Premenopausal			647 (57.9)	1055 (59.1)	0.108
	Postmenopausal			471 (42.1)	731 (41.9)	
	Unknown			20	148	
Family history of breastcancer (%)	No			1,044 (92.5)	1,301 (97.3)	4.6×10^−8^
	Yes			84 (7.5)	36 (2.7)	
	Unknown			10	97	
Histology (%)	DCIS			164 (14.4)	N.A.	N.A.
	Others			47 (4.1)	N.A.	N.A.
	IDC			927 (81.5)	N.A.	N.A.
		ER	Negative	231 (29.7)		
			Positive	548 (70.3)		
			Unknown	148		
		PR	Negative	273 (35.1)		
			Positive	504 (64.9)		
			Unknown	150		
		HER2	Negative	645 (82.9)		
			Positive	133 (17.1)		
			Unknown	149		
		Subtype	Luminal-like	496 (63.8)		
			HER2+	133 (17.2)		
			Triple-negative	148 (19.0)		
			Unknown	150		
		Lymph nodes	Negative	411 (57.1)		
			Positive	309 (42.9)		
			Unknown	207		
		Size	T1	414 (51.4)		
			T2	353 (43.8)		
			T3-4	39 (4.8)		
			Unknown	121		

DCIS, ductal carcinoma in situ; IDC, invasive ductal carcinoma; N.A., not applicable.

We also validated our results in another independent population with mainly familial/early-onset breast cancer cases. Since 2000, FUSCC has conducted a multi-center hospital-based gene mutation screening project in order to gain a full understanding of the contribution of germ-line mutations of high-penetrance genes to hereditary and early-onset breast cancer in the Han Chinese population [13]. The eligibility criteria have been described elsewhere [13]. All of the selected familial cases had been tested for BRCA1/2, BRIP1, and PALB2 germline mutations and no deleterious changes were found. Among all the recruited patients, we screened the candidates for the validation set using following criteria: 1, genetically unrelated Han Chinese women living in Shanghai City and its surrounding areas; 2, the pathology of tumor having been confirmed in the Department of Pathology of our hospital; 3, having spare and high quality DNA samples (most were available between 2006 and 2009) for genotyping. Finally, we selected 294 patients recruited between 2006 and 2009 as cases of the second set, 218 of them with available ER, PR, and HER2 status. The current study was approved by the Ethics Committee of FUSCC and all patients provided written informed consent. All clinical investigation had been conducted according to the principles expressed in the Declaration of Helsinki.

### Genotyping

Genomic DNA was extracted from the blood leukocytes of the participants using Gentra’s PureGene DNA Purification Kit (Gentra systems, USA). Genotyping was done using the 12-plex SNPstream system (Beckman Coulter, USA) at the Chinese National Human Genome Center at Shanghai. Primers and probe were: up, AAGATCCACTGGGCCAAG; low, AGGCAGCCTTTGATGGAA; probe, GCGGTAGGTTCCCGACATATGGCCAGGGGTGGTGTTGGGACAGCT. To ensure the reliability of the results, operators performing the genotyping assays were unaware of the disease status of each sample, and each batch of samples contained at least one positive control consisting of DNA samples with known genotype and two negative controls of pure water.

### Bioinformatic Analysis

TargetScan Human 5.2 (http://www.targetscan.org/) and MicroCosm Targets Version 5 (http://www.ebi.ac.uk/enright-srv/microcosm/htdocs/targets/v5/) were employed to predict the targets of miR-608. RNAfold (http://rna.tbi.univie.ac.at/) was used to calculate the secondary structure of miR-608 stem-loop sequence based on minimum free energy (MFE). RNAhybrid (http://bibiserv.techfak.uni-bielefeld.de/rnahybrid/) was used to evaluate the affinity of variant miR-608 and ancestral miR-608 to predicted targets, respectively.

### Power Analysis

The program Quanto (http://hydra.usc.edu/gxe) was used to estimate the statistical power. The variant allele frequency of miR-608 (about 60% according to our genotyping results), odds ratio (1.5 or 1.8), incidence of breast cancer in the studied population (25 in 100,000 in Shanghai, China), and sample sizes were taken as the parameters. For the first set, there were 1,138 overall cases, 927 IDC, 133 IDC with HER2+ subtype, accompanied by 1,434 controls. In the recessive model, the sample sizes had 99.9%, 99.5%, and 60% power to detect allele with OR of 1.5, and had 99.9%, 99.9%, and 89% power to detect allele with OR of 1.8, for the overall cases, IDC, and HER2+ cases, respectively. In the additive model, the sample sizes had 99.9%, 99.9%, and 85% power to detect allele with OR of 1.5, and had 99.9%, 99.9%, and 99.0% power to detect allele with OR of 1.8, for the overall cases, IDC, and HER2+ cases, respectively. For the second set, there were 31 IDC with HER2+ subtype, accompanied by 500 controls. In the recessive model, the sample sizes had only 20% power to detect allele with OR of 1.5, and had 35% power to detect allele with OR of 1.8, for the HER2+ cases. The power would be much higher if we combined the first and the second set together. All the tests of power calculation were two-sided.

### Statistical Analysis

Comparison between groups used χ^2^ test for categorical variables. Student’s t-test and Kruskal-Wallis test were used to compare continuous variables among two and more than two groups, respectively. Hardy-Weinberg equilibrium (HWE) was tested by χ^2^ tests. The multiple comparison P-values were corrected by Bonferroni correction. Odds ratio (OR) adjusted for age, age at menarche, menopause status, body mass index (BMI) and family history of breast cancer, along with 95% confidence interval (CI), were determined by logistic regression. A two-sided P-value ≤0.05 was considered statistically significant. Statistical analysis was performed using STATA v.10.0 and SPSS v.12.0.

## Results

In both study sets, genotype distributions of rs4919510:C>G in the controls was in agreement with HWE. The frequency of variant G-allele was about 57% in this study, in consistent with genotyping data in NCBI-dbSNP and HapMap database of Chinese population. In the first set, there was no association between rs4919510:C>G and breast cancer risk either in the overall cases or in the IDC cases (**Table S1**). In the sub-analysis according to the IHC-based breast cancer subtypes (**Table 2**), although we did not observe any significant relationship between rs4919510:C>G and luminal-like or triple-negative subtype, a remarkable increase in risk of HER2+ subtype (n = 133) was found in women carrying variant genotypes (CG/GG) in a dose-effect manner (Bonferroni corrected P of 3.6×10^−3^ for trend, and of 9.3×10^−4^ for heterogeneity). Variant G-allele was the risk allele (OR = 1.62; 95% CI, 1.23−2.15) compared with its ancestral C-allele. **Table 3** shows the results of multivariate analysis. rs4919510:C>G was independently related to HER2+ breast cancer risk in the dominant model (OR = 2.10, 95% CI, 1.15–3.82), recessive model (OR = 1.97, 95% CI, 1.34−2.90), as well as additive model (CG vs. CC, OR = 1.63 with 95% CI of 0.87−3.08; GG vs. CC, OR = 2.87 with 95% CI of 1.52−5.42; overall P = 0.001).

**Table 2:** Associations between rs4919510:C>G and breast cancer subtypes in the IDC cases in the first set.

rs4919510		Controls (n = 1,434)		Luminal-like (n = 496)		OR(95% CI)	P#	HER2+ (n = 133)		OR(95% CI)	P#	P*	Triple-negative (n = 148)		OR(95% CI)	P#
		n	%	n	%			n	%				n	%		
Additive model	CC	277	19.5	86	17.7	Ref.	0.26	16	12.0	Ref.	1.2×10^−3^	3.6×10^−3^	26	17.9	Ref.	0.89
	CG	684	48.3	255	52.6	1.20(0.90–1.61)		54	40.6	1.37(0.75–2.60)	3.1×10^−4&^	9.3×10^−4&^	72	49.7	1.12(0.69–1.87)	
	GG	456	32.2	144	29.7	1.02(0.74–1.40)		63	47.4	2.39(1.33–4.52)			47	32.4	1.10(0.65–1.89)	
Dominant model	CC	277	19.5	86	17.7	Ref.	0.38	16	12.0	Ref.	0.034	0.102	26	17.9	Ref.	0.64
	CG+GG	1140	80.5	399	82.3	1.13(0.86–1.49)		117	88.0	1.78(1.03–3.26)			119	82.1	1.11(0.71–1.81)	
Recessive model	CC+CG	961	67.8	341	70.3	Ref.	0.31	70	52.6	Ref.	3.9×10^–4^	1.2×10^–3^	98	67.6	Ref.	0.95
	GG	456	32.2	144	29.7	0.89(0.71–1.12)		63	47.4	1.90(1.30–2.76)			47	32.4	1.01(0.69–1.47)	
Allele	C	1238	43.7	427	44.0	Ref.	0.85	86	32.3	Ref.	3.4×10^–4^	1.0×10^–3^	124	42.8	Ref.	0.76
	G	1596	56.3	543	56.0	0.99(0.85–1.15)		180	67.7	1.62(1.23– 2.15)			166	57.2	1.04(0.81–1.34)	

All P-values for comparisons between breast cancer subtypes and controls. Some samples fail in genotyping. Ref., reference; OR, odds ratio; CI, confidence interval.

# P for heterogeneity.

& P for trend.

* P values after Bonferroni correction (by ×3).

**Table 3:** Multivariate analysis of risk for HER2-positive breast cancer of 1,567 subjects in the first set.

Characteristics		P	OR	95% CI
Age (continuous)#		0.26	0.98	0.95 to 1.01
Age at menarche (continuous)#		0.0004	0.81	0.72 to 0.91
Menopausal status	Pre. vs. Post.	0.042	1.91	1.02 to 3.57
BMI (continuous)#		0.009	1.09	1.02 to 1.17
Family history of breast cancer	No vs Yes	0.003	3.14	1.49 to 6.65
Genotype of rs4919510 in miR-608	Additive*			
	CC		1 (Reference)	
	CG	0.13	1.63	0.87 to 3.08
	GG	0.001	2.87	1.52 to 5.42
	Dominant (CC versus CG+GG)	0.016	2.10	1.15 to 3.82
	Recessive (CC+CG versus GG)	0.001	1.97	1.34 to 2.90

BMI, body mass index; OR, odds ratio; CI, confidence interval.

* the overall P value is 0.001 for the additive model.

# for continuous variables, younger age at menarche and higher BMI are risk.

OR and 95% CI calculated by logistic regression, adjusted for age, age at menarche, menopausal status, BMI, and family history of breast cancer. CC genotype is as reference in the additive and dominant model, CC+CG genotype as reference in the recessive model. Additive model, dominant model, and recessive model are tested respectively. The P-values and ORs with 95% CIs of co-variables are from logistic regression with additive model of rs4919510.

**Table 4:** Associations between rs4919510:C>G and HER2-positive breast cancer subtype in the IDC cases in combined sets (n = 3,366).

rs4919510		The Second Set (control, n = 500; cases, n = 294)						Combined Sets (controls, n = 1,934; cases, n = 1,432)					
		Ctls(n = 500)		HER2+ Cases (n = 31)		OR	P#/P*	Ctls (n = 1,934)		HER2+ Cases (n = 164)		OR	P#/P*
		n	%	n	%			n	%	n	%		
Additive	CC	77	15.7	2	6.5	Ref.	0.06/0.18	354	18.6	18	11.0	Ref.	1.1×10^–4^/3.3×10^−4^
	CG	230	46.8	11	35.5	1.84(0.39−17.43)	0.02&/0.06&	914	47.9	65	39.6	1.42(0.83−2.43)§	3.2×10^–5&^/9.6×10^–5&^
	GG	184	37.5	18	58.0	3.77(0.86−34.14)		640	33.5	81	49.4	2.49(1.45–4.48)§	
Dominant	CC	77	15.7	2	6.5	Ref.	0.16/0.48	354	18.6	18	11.0	Ref.	0.014/0.042
	CG+GG	414	84.3	29	93.5	2.70(0.66−23.76)		1554	81.4	146	89.0	1.88(1.14−3.12)§	
Recessive	CC+CG	307	62.5	13	41.9	Ref.	0.02/0.06	1268	66.5	83	50.6	Ref.	3.5×10^–5^/1.1×10^–4^
	GG	184	37.5	18	58.1	2.31(1.04−5.25)		640	33.5	81	49.4	1.97(1.43−2.72)§	
Allele	C	384	39.1	15	24.2	Ref.	0.02/0.06	1622	42.5	101	30.8	Ref.	3.6×10^–5^/1.1×10^−4^
	G	598	60.9	47	75.8	2.01(1.09−3.93)		2194	57.5	227	69.2	1.66(1.30−2.14)	

Ctls controls; IDC, invasive ductal carcinoma.

# P for heterogeneity.

& P for trend.

* P values after Bonferroni correction (by ×3).

§ OR adjusted for study cohort.

**Figure 1:**
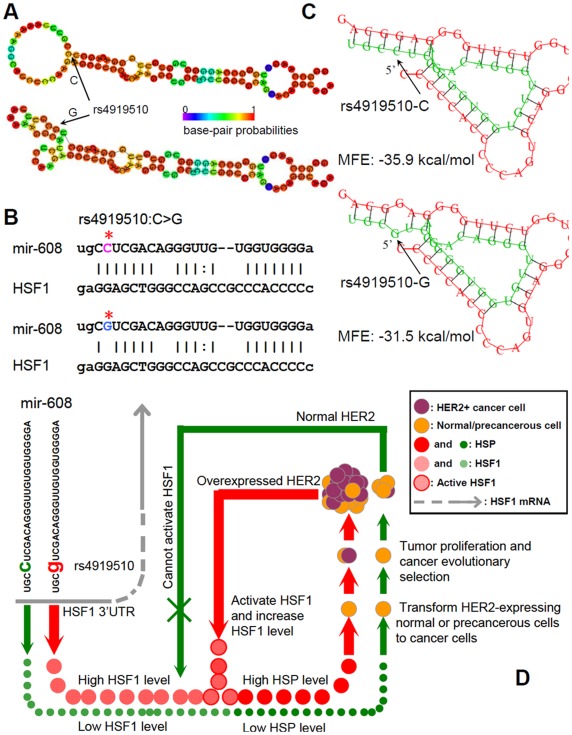
Bioinformatics prediction of rs4919510 within miR-608 and schematic representation of potential pathological mechanism of rs4919510 in HER2-positive breast cancer development. A, the predicted secondary structure of human miR-608 stem-loop sequence (100 bp) by RNAfold, with either the C- allele or G-allele. For ancestral form with C-allele, the optimal secondary structure with a minimum free energy (MFE) of −32.20 kcal/mol. The frequency of the MFE structure in the ensemble is 0.53%, and the ensemble diversity is 13.52. For the variant form, the optimal secondary structure with the same MFE of −32.20 kcal/mol. The frequency of the MFE structure is 0.33%, and the ensemble diversity is 14.09. The structure plot shows that variant G-allele strongly interfere with loop forming. B, HSF1 is a predicted target of miR-608 and rs4919510:C>G is just located at the complementary sequence. Asterisk indicates the polymorphic site. The plot of predicted duplex formation is from MicroCosm Targets website. C, Predicted secondary structure of duplex formation between miR-608 and 3′UTR of HSF1 by RNAhybrid. rs4919510:C>G changes the MFE, with ancestral form of −35.9 kcal/mol and variant form of −31.5 kcal/mol. D, schematic representation of speculated pathological mechanism of rs4919510 in HER2-positive breast cancer development. In this model, miR-608 harbouring the rs4919510-C allele (ancestral form) is set as the baseline condition. The C-to-G substitution might weaken the suppression of HSF1 mRNA by miR-608, leading to relatively high expression of HSF1 protein and, in turn, up-regulating HSPs and facilitating HER2-expressing normal or precancerous breast cells to transform cancer cells. HSPs also promote tumor cell proliferation. After cancer evolutionary selection, HER2-overexpressed or HER2-amplified tumor is formed since HER2+ cells are easy to survive under HSF1/HSPs stimulating. Moreover, overexpression of HER2 would activate HSF1 and promote HSF1 protein synthesis, further upregulating HSPs and facilitating tumorigenesis and development of HER2+ breast cancer.

We subsequently analyzed the influence of rs4919510:C>G on the breast tumor development. Analogously, rs4919510:C>G was unrelated to either tumor size (reflecting local tumor proliferation) or lymph nodes status (reflecting tumor dissemination and metastasis potentials) in the overall IDC, luminal-like subgroup, or triple-negative subgroup (**Table S2**). However, there was an incremental risk of high stage of tumor size (T2-4 vs T1) in patients carrying variant homozygous genotypes of rs4919510:C>G in the HER2+ cases (P for heterogeneity = 0.017, P for trend = 0.004). If we treat the tumor size as a continuous variable, we still found that patients harbouring GG genotype had larger tumor sizes compared with those carrying CC or CG (Overall P for Kruskal-Wallis test = 0.006; P-values for Dunn’s Multiple Comparison test of CC vs GG or CG vs GG were all <0.05, **Figure S1**).

To validate our findings in the first set, we performed another independent case-control study involving 294 familial/early-onset breast cancer cases and 500 hospital-based cancer-free controls. Similar to the first study, there was no fundamentally different result in the second set, which showed that GG genotype was associated with an increased risk of HER2-positive breast cancer, with unadjusted P value of 0.02 and corrected P value of 0.06. The associations were more significant when the two studies were combined together (total number of HER2+ cases were 164), with a crude P value of 3.6×10^–5^ for the G allele. After conservative Bonferroni correction, rs4919510:C>G was still significantly associated with increased risk of HER2-positive breast cancer (**Table 4**).

In order to reveal the potential genetic and molecular mechanism of epidemiological observation, we subsequently conducted bioinformatic analysis. First, we predicted the secondary structure of variant and ancestral miR-608 stem-loop sequence, respectively. Though the optimal secondary structure of the two forms had the same MFE of –32.2 kcal/mol, the free energy of the thermodynamic ensemble, frequency of MFE structure in the ensemble, and ensemble diversity were all changed (**Figure 1A**). Using MicroCosm Targets and TargetScan tools, 963 and 189 targets of miR-608 were identified respectively. We scrutinized all the candidate transcripts and found nine targeting transcripts closely related to breast carcinogenesis and progression according to current literature. Among them, miR-608 might bind to the 3′ untranslated regions (3′UTR) at the complementary sequence containing polymorphic site of rs4919510:C>G in two transcripts, one was heat shock transcription factor-1 (HSF1) (**Figure 1B**) and the other was lymphocyte-specific protein-1 (LSP1). Since the current literature suggested an association of HER2+ breast cancer with HSF1 but not with LSP1, we further analyzed the differential affinity of variant and ancestral miR-608 to the 3′UTR of HSF1. The MFE of ancestral miR-608 binding to HSF1 was −35.9 kcal/mol, while that of variant form was −31.5 kcal/mol, indicating a lower affinity of variant miR-608 to the binding sites in HSF1 3′UTR (**Figure 1C**).

## Discussion

In this study, we for the first time reported that variant genotype of rs4919510:C>G located in mature miR-608 was associated with significantly increased risk of HER2+ breast cancer but not other subtypes. Although the significant association between rs4919510:C>G and HER2+ breast cancer was observed in the stratified population, the power analysis demonstrated that the current sample size has 85% power to identify allele with OR of 1.5 in the additive model. Univariate and multivariate analyses consistently showed a risk role of rs4919510 G-allele for HER2+ subtype. More importantly, we validated the findings in a second independent population with a borderline significance. It was likely that the relatively small sample size of the second study made a borderline significant. When we combined the two sets together, G allele and GG genotype were more significantly associated with increased risk of HER2-positive breast cancer even after conservative Bonferroni correction. We believe our observed association is likely true rather than false positive.

To identify breast cancer-related mRNA targeted by miR-608, we surveyed the predicted transcripts. Among them, two transcripts (HSF1 and LSP1) were predicted to bind miR-608 at the polymorphic site of rs4919510:C>G. HSF1 is of particular interests because it is recently proven to be required for HER2-induced tumorigenesis and HER2-expressing cell proliferation. The potential mechanism is likely that HSF1 maintains levels of heat shock proteins (HSPs) such as HSP72 and HSP27 [14]. On the other hand, HER2 can activate HSF1 by increasing HSF1 trimer formation and promoting HSF1 protein synthesis [15]. It seems there is a synergistic loop between HER2 and HSF1, and slight changes in HSF1 level (regulated by rs4919510:C>G in miR-608) might be amplified by the loop, resulting in more significant alterations in HSF1/HSPs level and consequently facilitating pathological outcomes such as HER2+ breast cancer transformation and proliferation (illustrated in **Figure 1D**). The outcomes of *in silico* analysis and theoretical deduction well explain the epidemiological observations. In addition, bioinformatic analysis has also showed LSP1 mRNAs might be targeted by miR-608 and rs4919510:C>G is exactly located at the binding sites. LSP1 is reported as a susceptibility locus of breast cancer in genome-wide association studies [16], [17]. However, it is not found to be associated with HER2+ subtype [18] and its exact role in HER2+ breast cancer development remains to be investigated.

The limitation of this study should be acknowledged. First, when we compared the controls from the first set with those from the second set, there was a significant difference between the two control population (in additive model, CC vs GC vs GG: P = 0.046; in recessive model, CC+GC vs GG: P = 0.032). These two sets were genotyped using the same genotyping platform and comparable technical procedure. Therefore, the observed difference might be caused by the population heterogeneity between the two control sets. The first control set was from a community-based breast cancer screening program, while the second set was from the hospital-based female population (main from the Department of Breast Surgery). Since most women came to our hospital for cancer screening as well as dealing with benign breast disease, the enrolled control women in the second set could have a higher prevalence of benign breast disease. Of note, HSF1, a predicted target of miR-608, has been proved to be associated with cell proliferation. It is reasonable to conjecture that the higher proportion of G-allele in the hospital-based controls compared with the community-based controls is probably due to the pathological effect of rs4919510:C>G on benign breast disease. In other word, rs4919510:C>G in miR-608 might participate in the whole procedure of normal cell proliferation, preneoplasia formation, and cancer initiation. Of course, this issue is beyond the current scope of this article and needs further investigation. Second, although we have investigated two study cohorts including reasonable sample sizes, due to rare frequency of HER2+ breast cancer, the sample size is quite small (in the first set, n = 133; in the second set, n = 31) for the investigation of HER2+ breast cancer specific risk effects. The power calculation for the second set also indicated that the validation samples had a low power of 20−35% to identify a true association between risk of HER2+ subtype breast cancer and rs4919510:C>G if we assumed the relative risk of G-allele at 1.5–1.8.

In summary, we identified that rs4919510:C>G in mature sequence of miR-608 may affect breast cancer risk and influence tumor proliferation. It is speculated that differential regulations of HSF1 mRNA by variant and ancestral miR-608 result in the differential HSF1 levels, leading to differential development of HER2+ breast cancer. Further replication studies of our findings with diverse ethnic groups and functional characterization of rs4919510:C>G variant in miR-608 are warranted.

## Supporting Information

Figure S1 Different pathological tumor size according to rs4919510:C>G genotype. Patients harbouring GG genotype had larger tumor sizes compared with those carrying CC and CG genotypes. P for overall Kruskal-Wallis test = 0.006. P-values for Dunn’s Multiple Comparison test of CC vs GG or CG vs GG were all <0.05.
(PPT)

Table S1 Associations between rs4919510:C>G and breast cancer risk in the overall population and in the IDC cases in the first set.
(DOC)

Table S2 Associations of rs4919510:C>G genotype with breast tumor size and lymph nodes status in HER2-positive IDC cases in the first set.
(DOC)
