# Supplementary figures and images for: Polymorphism rs4919510:C>G in Mature Sequence of Human MicroRNA-608 Contributes to the Risk of HER2-Positive Breast Cancer but Not Other Subtypes
Source: PLoS One. 2012 May 7;7(5):e35252. doi: 10.1371/journal.pone.0035252 (PMC3346742; doi:10.1371/journal.pone.0035252)

## Slide 1
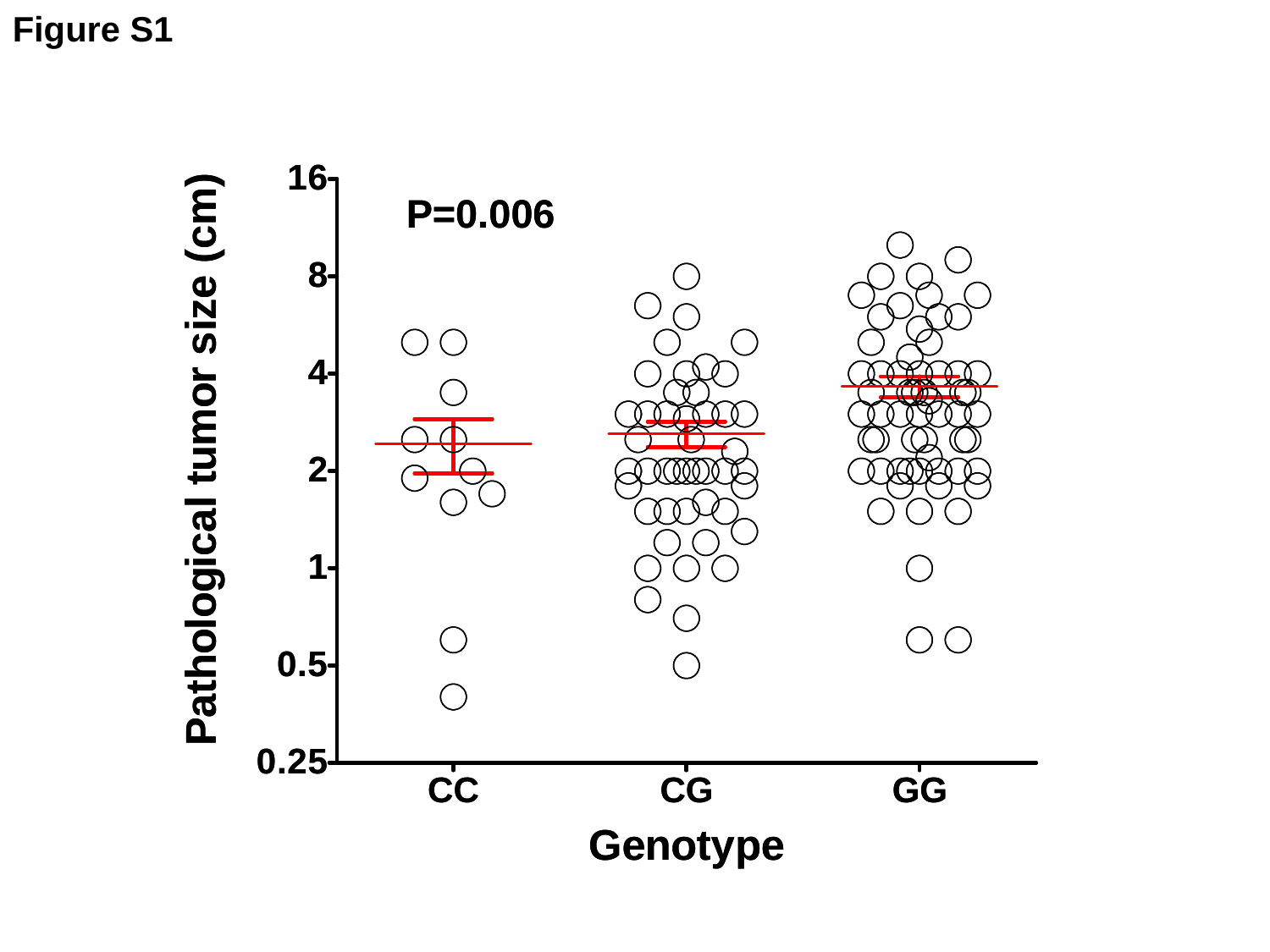

Figure S1

Supplement: Figure S1 — Different pathological tumor size according to rs4919510:C>G genotype. Patients harbouring GG genotype had larger tumor sizes compared with those carrying CC and CG genotypes. P for overall Kruskal-Wallis test = 0.006. P-values for Dunn’s Multiple Comparison test of CC vs GG or CG vs GG were all <0.05. (PPT) [file pone.0035252.s001.ppt]
