# Supplementary material for: Polymorphism rs4919510:C>G in Mature Sequence of Human MicroRNA-608 Contributes to the Risk of HER2-Positive Breast Cancer but Not Other Subtypes
Source: PLoS One. 2012 May 7;7(5):e35252. doi: 10.1371/journal.pone.0035252 (PMC3346742; doi:10.1371/journal.pone.0035252)
Supplement: Table S1 — Associations between rs4919510:C>G and breast cancer risk in the overall population and in the IDC cases in the first set. (DOC) [file pone.0035252.s002.doc]

**Supplemental Tables**

**Table S1**

**Associations between rs4919510:C>G and breast cancer risk in the overall population and in the IDC cases in the first set.**

| **rs4919510** | | **Controls (n=1,434)** | |  | **All cases (n=1,138^*^)** | | **P (all cases vs controls)** | **IDC cases (n=927^*^)** | | **P (IDC vs controls)** |
| --- | --- | --- | --- | --- | --- | --- | --- | --- | --- | --- |
|  |  | **n** | **%** |  | **n** | **%** |  | **n** | **%** |  |
| Additive model | CC | 277 | 19.5 |  | 192 | 17.2 | 0.27 | 156 | 17.1 | 0.35 |
|  | CG | 684 | 48.3 |  | 545 | 48.7 |  | 452 | 49.7 |  |
|  | GG | 456 | 32.2 |  | 381 | 34.1 |  | 302 | 33.2 |  |
| Dominant model | CC | 277 | 19.5 |  | 192 | 17.2 | 0.13 | 156 | 17.1 | 0.15 |
|  | CG+GG | 1140 | 80.5 |  | 926 | 82.8 |  | 754 | 82.9 |  |
| Recessive model | CC+CG | 961 | 67.8 |  | 737 | 65.9 | 0.31 | 608 | 66.8 | 0.61 |
|  | GG | 456 | 32.2 |  | 381 | 34.1 |  | 302 | 33.2 |  |
| Allele | C | 1238 | 43.7 |  | 929 | 41.5 | 0.13 | 764 | 42.0 | 0.25 |
|  | G | 1596 | 56.3 |  | 1307 | 58.5 |  | 1056 | 58.0 |  |

^*^Some samples fail in genotyping.
