# Supplementary material for: Polymorphism rs4919510:C>G in Mature Sequence of Human MicroRNA-608 Contributes to the Risk of HER2-Positive Breast Cancer but Not Other Subtypes
Source: PLoS One. 2012 May 7;7(5):e35252. doi: 10.1371/journal.pone.0035252 (PMC3346742; doi:10.1371/journal.pone.0035252)
Supplement: Table S2 — Associations of rs4919510:C>G genotype with breast tumor size and lymph nodes status in HER2-positive IDC cases in the first set. (DOC) [file pone.0035252.s003.doc]

**Table S2**

**Associations of rs4919510:C>G genotype with breast tumor size and lymph nodes status in HER2-positive IDC cases in the first set.**

| **Characteristics** | | | **rs4919510 genotype** | | | | | | **P** |
| --- | --- | --- | --- | --- | --- | --- | --- | --- | --- |
|  |  |  | **CC (n=128)** | **%** | **CG (n=381)** | **%** | **GG (n=254)** | **%** |  |
| Luminal-like | Tumor size | T1 | 47 | 56.6 | 134 | 55.1 | 72 | 52.2 | 0.78^*^ |
|  |  | T2-4 | 36 | 43.3 | 109 | 44.9 | 66 | 47.8 | 0.50^#^ |
| HER2+ | Tumor size | T1 | 6 | 54.5 | 25 | 54.4 | 17 | 28.3 | 0.017^*^ |
|  |  | T2-4 | 5 | 45.5 | 21 | 45.6 | 43 | 71.7 | 0.004^#^ |
| Triple-negative | Tumor size | T1 | 12 | 48.0 | 31 | 43.7 | 19 | 44.2 | 0.93^*^ |
|  |  | T2-4 | 13 | 52.0 | 40 | 56.3 | 24 | 55.8 | 0.92^#^ |
|  |  |  |  |  |  |  |  |  |  |
| Luminal-like | Lymph nodes | Negative | 38 | 54.3 | 124 | 58.5 | 63 | 53.4 | 0.63^*^ |
|  |  | Positive | 32 | 45.7 | 88 | 41.5 | 55 | 46.6 | 0.76^#^ |
| HER2+ | Lymph nodes | Negative | 9 | 60.0 | 24 | 52.2 | 22 | 45.8 | 0.60^*^ |
|  |  | Positive | 6 | 40.0 | 22 | 47.8 | 26 | 54.2 | 0.32^#^ |
| Triple-negative | Lymph nodes | Negative | 9 | 52.9 | 37 | 59.7 | 22 | 53.7 | 0.79^*^ |
|  |  | Positive | 8 | 47.1 | 25 | 40.3 | 19 | 46.3 | 0.87^#^ |

^*^ P for heterogeneity

^#^ P for trend
